# Auto Regressive Moving Average (ARMA) Modeling Method for Gyro Random Noise Using a Robust Kalman Filter

**DOI:** 10.3390/s151025277

**Published:** 2015-09-30

**Authors:** Lei Huang

**Affiliations:** Automation Department, Nanjing Forestry University, 159 Longpan Road, Nanjing 210037, China; E-Mail: huanglei@njfu.edu.cn

**Keywords:** random noise modeling, robust Kalman filtering, ARMA modeling

## Abstract

To solve the problem in which the conventional ARMA modeling methods for gyro random noise require a large number of samples and converge slowly, an ARMA modeling method using a robust Kalman filtering is developed. The ARMA model parameters are employed as state arguments. Unknown time-varying estimators of observation noise are used to achieve the estimated mean and variance of the observation noise. Using the robust Kalman filtering, the ARMA model parameters are estimated accurately. The developed ARMA modeling method has the advantages of a rapid convergence and high accuracy. Thus, the required sample size is reduced. It can be applied to modeling applications for gyro random noise in which a fast and accurate ARMA modeling method is required.

## 1. Introduction

In strapdown inertial navigation systems (SINS), the ARMA model, which is one of the three time series models, is usually used to model or compensate for optic gyro random errors [1]. After the ARMA model is established, Kalman filtering or other filters are usually employed for noise reduction [2,3]. However, Kalman filter itself is seldom used to model the gyro random error anymore. The conventional ARMA modeling methods for gyro random noise, such as least square method, moment estimation method, and maximum likelihood method, all must first determine the model order before the ARMA model parameters are estimated. The order determination processes are complex, and the parameter estimations of the conventional methods require a large sample size and have a slow convergence speed [4,5,6,7]. Hence, they cannot be applied to applications in which a fast and accurate ARMA modeling method for gyro random noise is required. To overcome these drawbacks, this paper developed a new ARMA modeling method for gyro random noise using a robust Kalman filtering. The developed modeling method does not require the complex model order determination. The order and the parameter estimates of the ARMA model can be identified simultaneously, quickly, and accurately by the developed method.

## 2. ARMA Modeling Method Using a Robust Kalman Filtering

To build the time series model (e.g., ARMA) of the gyro random noise, there are three steps to be followed after obtaining the raw gyro noise data [8,9,10]: (1) Randomness and stationarity tests. If the gyro’s raw noise data do not meet the requirements of stationary random noise, we can usually obtain the gyro stationary random noise after first or second order difference. (2) Selection of the suitable time series model according to auto-correlation and partial correlation characteristics of FOG random noise. (3) Parameter estimation of the time series model.

### 2.1. Randomness and Stationarity Test and Analysis of the Auto-Correlation and Partial Correlation Characteristics

**Figure 1:**
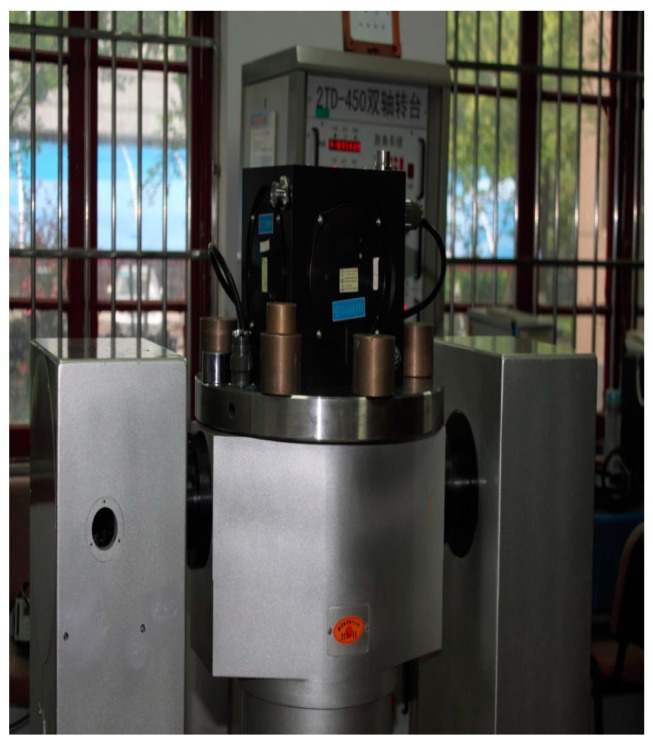
Experimental picture.

**Figure 2:**
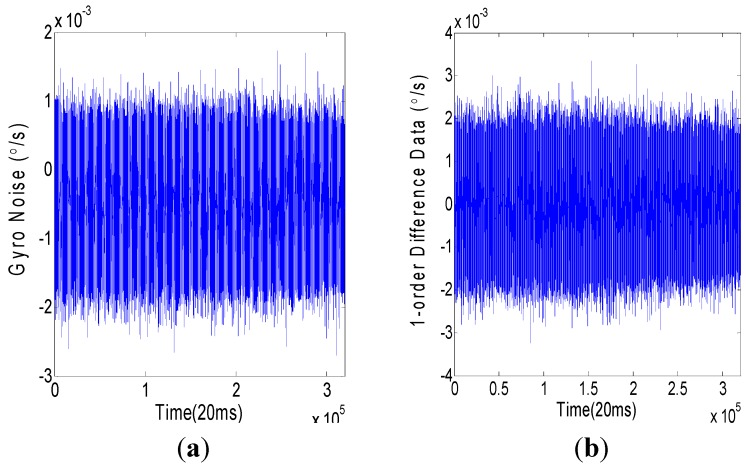
Random noise of VG951 FOG. (**a**) Original data (°/s). (**b**) First-order difference of random noise.

A 6400 s ground experiment was employed to collect the output noise data of a VG951 (Fizoptika, Arzamas, Russia) fiber optic gyro (FOG). The experimental picture is shown in Figure 1. The sampling rate was 50 Hz. The data unit was °/s. The output noise data (*x*-axis, 320,000 samples) of the VG951 FOG is shown in Figure 2a. After the randomness test, some fixed terms were found in the noise data. By a first-order difference, these fixed terms were removed, and FOG random noise data that satisfied the stationary random requirements were obtained and are shown in Figure 2b.

**Figure 3:**
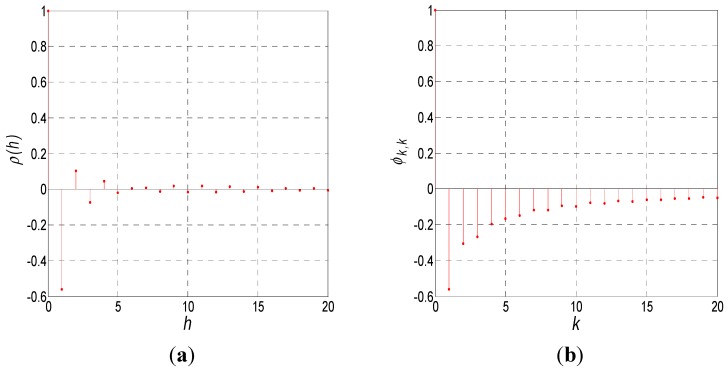
Self-correlation and partial correlation coefficient functions of VG951 random noise: (**a**) Self-correlation; (**b**) Partial correlation.

The auto-correlation coefficient function *p*(*h*) and partial correlation coefficient function *φ_k,k_* of the FOG random noise data are shown in Figure 3. As is seen in Figure 3, both the auto-correlation and partial correlation coefficients decay slowly. According to the time series theory, this type of data is applicable to the ARMA model. However, the order of the ARMA is unknown.

### 2.2. ARMA Modeling Method Using a Robust Kalman Filter

The ARMA model is defined as:

(1) $$z(k)=\sum_{i=1}^{p}a_{i}z(k-i)+\varepsilon (k)+\sum_{j=1}^{q}b_{j}\varepsilon (k-j)$$

where *ε*(*k*) is a zero mean and unknown variance white noise.

#### 2.2.1. State Equation and Measurement Equation

First, we suppose that the model order of the VG951 random noise is ARMA (1,1):

(2) $$z(k)=a_{1}z(k-1)+b_{1}\varepsilon (k-1)+\varepsilon (k)$$

The parameters of ARMA (1,1) are taken as a state argument: *X*(*k*) = [*a*_1_(*k*), *b*_1_(*k*)]^T^. Thus, Equation (2) can be regarded as a measurement equation:

(3) z(k)=H(k)X(k)+ε(k)

where *H* is the measurement matrix *H(k)*=[*z*(*k*−1), *ε*(*k*−1)]. Because the value of white noise *ε*(*k* − 1) cannot be obtained, *ε*(*k* − 1) will be replaced by its estimate $\hat{\varepsilon }(k-1)$ during the Kalman filtering process. Thus, the measurement matrix *H*(*k*) will be replaced by its estimate $\hat{H}(k)$, and Equation (3) can be rewritten as:

(4) $$z(k)=\hat{H}(k)X(k)+v(k)$$

where *v*(*k*) is virtual measurement noise caused by white noise *ε*(*k*) and the measure matrix error Δ*H*(*k*). These are expressed as:

(5) $$\left\{ \begin{array}{l} \hat{H}(k)=\left[z(k-\text{1}),\hat{\varepsilon }(k-\text{1})\right]\\ v(k)=\varepsilon (k)+[H(k)-\hat{H}(k)]X(k)=\varepsilon (k)+\text{Δ}H(k)X(k) \end{array}\right.$$

Obviously, the characteristic of Δ*H*(*k*)*X*(*k*) is an unknown time variable, and Δ*H*(*k*) is small compared to *H*(*k*). Hence, the virtual measurement noise *v*(*k*) is approximately unknown time-variable white noise. When the ARMA mode is stable, the estimates of the state argument by Kalman filtering should converge to the real values. In addition, the ARMA parameter estimates will not change with the new FOG random noise sample if enough sample size is given. These are ${\hat{a}}_{1}(k+1)={\hat{a}}_{1}(k)=a_{1}$ and ${\hat{b}}_{1}(k+1)={\hat{b}}_{1}(k)=b_{1}$, where “^” represents the estimate. Thus, the system state equation is:

(6) X(k+1)=X(k)

From Equation (6), we know that the state transition matrix $\text{Φ}=I_{2}=\left[\begin{array}{l} \;1\;0\\ \;0\;1 \end{array}\right]$ and the system noise *w*(*k*) = 0. Hence:

(7) $$\left\{ \begin{array}{l} q_{k}=Ew_{k}=0,\;Q_{k}=\mathrm{cov}[w_{k},w_{j}]=0\\ Ev_{k}=r_{k},\;\mathrm{cov}[v_{k},v_{j}]=R_{k}\delta_{kj}\\ \;E[w_{k}v_{j}^{T}]=0 \end{array}\right.$$

Equation (4) is the measurement equation. Equation (6) is the state equation. System noise and measurement noise characteristics are given in Equation (7). As stated, the measurement noise *v*(*k*) is unknown time-variable white noise. An unknown time-variable estimator should be used to estimate the mean and variance of the measurement noise [11,12,13,14]:

(8) $$\left\{ \begin{array}{l} {\hat{r}}_{k}=(1-d_{k-1}){\hat{r}}_{k-1}+d_{k-1}(z_{k}-H_{k}{\hat{x}}_{k|k-1})\\ {\hat{R}}_{k}=(1-d_{k-1}){\hat{R}}_{k-1}+d_{k-1}(\varepsilon_{k|k-1}\varepsilon_{k|k-1}^{T}-H_{k}P_{k|k-1}H_{k}^{T}), \end{array}\right.$$

where *ε*_*k|k*−1_ can be calculated by Equation (12). The corresponding Kalman filter is usually called a robust Kalman filter [11].

#### 2.2.2. Parameter Estimation Using a Robust Kalman Filter

As stated, the state and measurement equations are given in Equations (4)–(6). System and measurement noise estimation equations are given in Equations (7) and (8). Thus, the ARMA (1,1) model parameter estimates can be obtained by the robust Kalman filter. The computing processes for the robust Kalman filter are listed as follows:

(9) $${\hat{x}}_{k|k-1}={\text{Φ}}_{k|k-1}{\hat{x}}_{k-1|k-1}={\hat{x}}_{k-1|k-1}$$

(10) $$P_{k|k-1}={\text{Φ}}_{k|k-1}P_{k-1|k-1}{\text{Φ}}_{k|k-1}^{T}+{\text{Γ}}_{k|k-1}Q_{k-1}{\text{Γ}}_{k|k-1}^{T}=P_{k-1|k-1}$$

(11) $$K_{k}=P_{k|k-1}+H_{k}^{T}{\left[H_{k}P_{k|k-1}H_{k}^{T}+{\hat{R}}_{k-1}\right]}^{-1}$$

(12) $$\varepsilon_{k|k-1}=z_{k}-H_{k}{\hat{x}}_{k|k-1}-{\hat{r}}_{k-1}$$

(13) $${\hat{x}}_{k|k}={\hat{x}}_{k|k-1}+K_{k}\varepsilon_{k|k-1}$$

(14) $$P_{k|k}=[I_{n}-K_{k}H_{k}]P_{k|k-1}$$

where the system noise mean *q_k_* = 0 and variance *Q_k_* = 0. The state-argument comprises the ARMA (1,1) parameters: *X* = [*a*_1_(*k*), *b*_1_(*k*)]*^T^*. The measurement matrix estimate $\hat{H}$ is calculated by Equation (5), and $\hat{\varepsilon }k$ in Equation (5) is calculated by Equation (12). The mean and variance estimates of the measurement noise are calculated by Equation (8). The initial values of the robust Kalman filter are set as: $\hat{X}$(0|0) = [[0.1, −0.1]*^T^*, *P*(0) = 100*I*_2_, *b* = 0.975, ${\hat{r}}_{k}(0)=0$, ${\hat{R}}_{k}(0)=0.01$.

**Figure 4:**
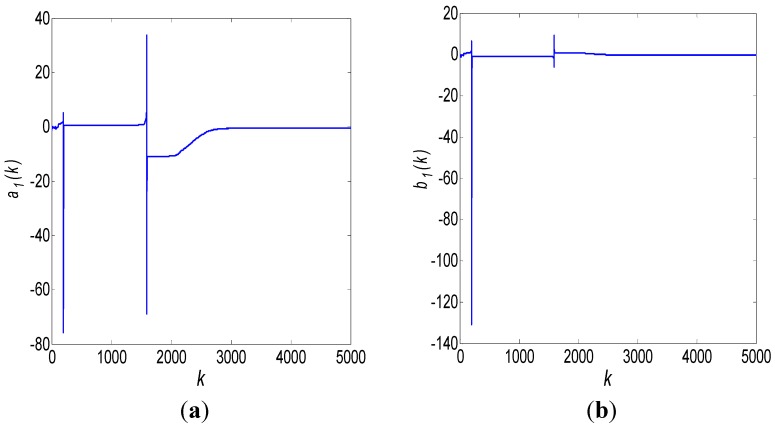
ARMA (1,1) modeling results using a robust Kalman filter: (**a**) *a*_1_ estimate; (**b**) *b*_1_ estimate.

After 3000 iterations, the results of robust Kalman filtering are shown in Figure 4:

Obviously, the Kalman filtering results shown in Figure 4 are morbid and divergent. This is because the order of the used ARMA model is incorrect, so the state argument *X* and the measurement matrix *H* are incorrect as well. Hence, we change the order of the ARMA model to ARMA (1,2):

(15) $$z(k)=a_{1}z(k-1)+b_{1}\varepsilon (k-1)+b_{2}\varepsilon (k-2)+\varepsilon (k)$$

**Figure 5:**
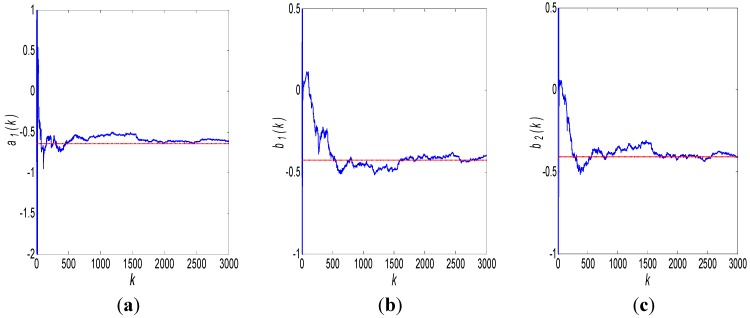
ARMA (1,2) modeling results using a robust Kalman filter: (**a**) *a*_1_ estimate, (**b**) *b*_1_ estimate, and (**c**) *b*_2_ estimate.

Thus, the state argument is changed to *X* = [*a*_1_(*k*),*b*_1_(*k*),*b*_2_(*k*)]*^T^*, and measurement matrix estimate $\hat{H}\left(k\right)=\left[z\left(k-\text{1}\right),\hat{\varepsilon }\left(k-\text{1}\right),\hat{\varepsilon }\left(k-2\right)\right]$. The initial values of the Kalman filter are set as $\hat{X}(0|0)$ = [0.1, −0.1, 0.5]*^T^*, *P*(0) = 100*I*_3_. The other parameters are held. The results of the robust Kalman filtering are shown by the (blue) solid curve in Figure 5a–c. The (red) dashed line in Figure 5 represents the results modeled by the conventional method of Maximum Likelihood using 6400 s data and 320,000 samples, which is:

*z*(*k*)= − 0.643*z*(*k* − 1) − 0.426*ε*(*k* − 1) − 0.389*ε*(*k* − 2) + *ε*(*k*) (16)

When the Kalman filter runs, a threshold *θ* is used to decide when the Kalman filtering iteration will be exited. If the differences between the maximum and minimum state argument estimates in ten runs are all consistently less than *θ*, the state argument estimates can be considered to be convergent. Thus, the Kalman filter will be stopped. In this test, we set: *θ*_*a*1_ = *θ*_*b*1_ = *θ*_*b*2_ = 0.001. The Kalman filter exits after 2230 runs, and the state-argument estimates converge to:

(17) $$\hat{X}(2230)=\left[\begin{array}{l} {\hat{a}}_{1}(2230)\\ {\hat{b}}_{1}(2230)\\ {\hat{b}}_{2}(2230) \end{array}\right]=\left[\begin{array}{l} -0.614\\ -0.410\\ -0.401 \end{array}\right]$$

Thus, the ARMA (1,2) model established by the robust Kalman filter is:

(18) z(k)=−0.614z(k−1)−0.410ε(k−1)−0.401ε(k−2)+ε(k)

If we model the same 2230 samples with the Maximum Likelihood method, the result is:

(19) z(k)=−0.539z(k−1)−0.578ε(k−1)−0.376ε(k−2)+ε(k)

Using the model of Equation (16) (Maximum Likelihood method, 320,000 samples) as a reference standard, the comparisons of Equations (18) and (19) with Equation (16) are shown in Table 1.

**Table 1:** Modeling results comparisons between different methods (*x*-axis).

Axis	Modeling Method	Sample Size	*a*_1_	*b*_1_	*b*_2_
*X*	Maximum Likelihood	320,000	−0.643	−0.426	−0.389
	Developed Method	2230	−0.614	−0.410	−0.401
	Maximum Likelihood	2230	−0.539	−0.578	−0.376

From Table 1, we can see that given the same small sample size (2230), the model established by the developed method (Equation (18)) are closer to the reference standard than that established by the conventional Maximum Likelihood method (Equation (19)). Therefore, the developed ARMA modeling method has better precision than the conventional Maximum Likelihood method under the condition of a small sample set.

**Figure 6:**
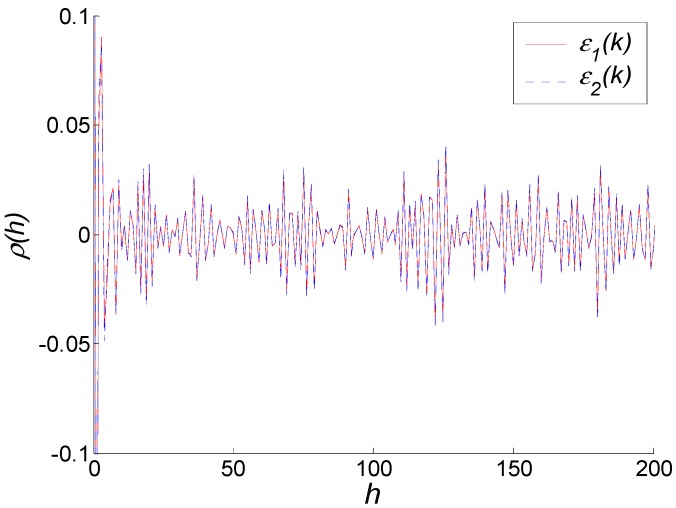
White noise test of two modeling methods.

It should be noted that on the finite sample size condition, the ARMA model established by different methods (e.g., Maximum Likelihood and moment estimation) using the same samples are not identical [15,16,17,18,19]. The small differences between the model established by the developed method using 2230 samples (Equation (16)) and the model established by the Maximum Likelihood method using 320,000 samples (Equation (18)) are in the allowable range. To prove this, a white noise test is employed to test the modeling efforts of different methods. In Figure 6, the solid line is the autocorrelation coefficient of white noise estimates *ε*_1_, which is obtained from Equation (18), and the dashed line is the autocorrelation coefficient of white noise estimates *ε*_2_, which is obtained from Equation (16). It can be seen that *ε*_1_ and *ε*_2_ coincide nearly and are both less than 0.1. Thus, both *ε*_1_, *ε*_2_ can be approximately considered as white noise. Then the modeling results of two modeling methods, *i.e.*, Equations (16) and (18), are both correct.

However, the developed method requires only 44.6 s of test time (2230 samples). Hence, the developed ARMA modeling method using the robust Kalman filter can estimate the ARMA model parameters correctly with a smaller sample size. To further validate the advantages of the developed ARMA modeling method, we modeled the *y*-axis and *z*-axis FOG random noise using the developed method and Maximum Likelihood method, respectively. This is feasible because both of the auto-correlation and the partial correlation coefficients for *y*-axis and *z*-axis random noise decay slowly. According to the time series theory, the *y*-axis and *z*-axis data are applicable to the ARMA model. The modeling results are listed in Table 2:

**Table 2:** Modeling result comparisons between different methods (*y*, *z*-axis).

Axis	Modeling Method	Sample Size	*a*_1_	*b*_1_	*b*_2_	*b*_3_
*Y*	Maximum Likelihood	320,000	−0.566	−0.431	−0.567	−0.136
	Developed Method	1367	−0.589	−0.428	−0.551	−0.123
	Maximum Likelihood	1367	−0.604	−0.417	−0.483	−0.182
*Z*	Maximum Likelihood	320,000	−0.638	−0.328	−0.465	-
	Developed Method	2073	−0.643	−0.359	−0.459	-
	Maximum Likelihood	2073	−0.534	−0.563	−0.409	-

**Figure 7:**
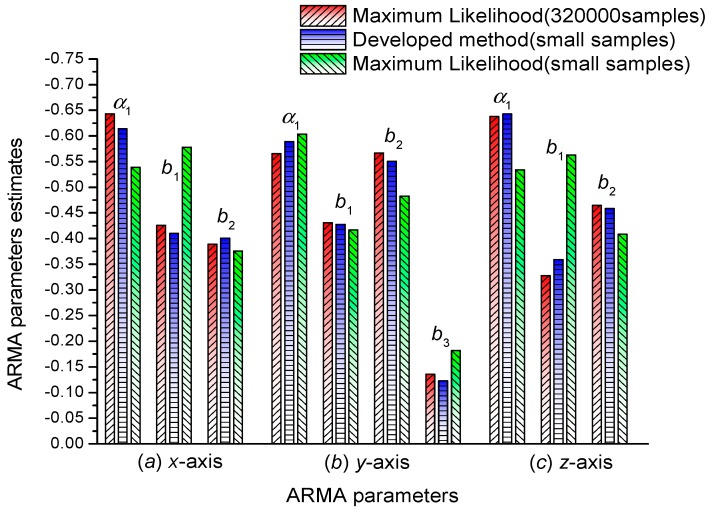
Comparison of ARMA modeling results using different methods.

First, we tried to employ ARMA (1,2) to model the *y*-axis and *z*-axis data. However, the Kalman filtering results for *y*-axis data using ARMA (1,2) model are divergent like the ones shown in Figure 4. Then, we employed ARMA (1,3) to model the *y*-axis data, and the corresponding Kalman filtering results converge correctly. Therefore, in Table 2 the ARMA model order for *y*-axis data is ARMA (1,3), and the ARMA model order for *z*-axis data is ARMA (1,2). To provide a clear comparison, the modeled results using different methods in Table 1 and Table 2 are shown in Figure 7.

From Figure 7, we can see that only small samples are required (*x*-axis: 2230, *y*-axis: 1367, and *z*-axis: 2073), and the modeling result using the developed method can obtain the same precision as the Maximum Likelihood method using 320,000 samples. Under the same condition of small samples (*x*-axis: 2230, *y*-axis: 1367, and *z*-axis: 2073), the developed method has obviously higher precision than that of the Maximum Likelihood method. Thus, the developed method has the advantages of fast convergence and high precision, and it is applicable to cases in which a fast and accurate modeling method is required.

## 3. Conclusions

Comparing with the conventional ARMA modeling methods, the developed method using the robust Kalman filtering can establish the ARMA model for FOG random noise correctly with a smaller sample size. During the modeling process, the developed method does not require the complex model order determination. The model order and parameter estimates can be determined simultaneously and quickly. In addition, when the new samples of FOG random noise are obtained, the robust Kalman filtering can update the ARMA model parameters through the new samples. The model established by the developed method can follow the characteristic variation of the FOG random noise quickly. Thus, the developed method can be applied to FOG noise modeling applications in which a fast ARMA modeling method is required.
